# Supplementary figures and images for: Cryptosporidium life cycle small molecule probing implicates translational repression and an Apetala 2 transcription factor in macrogamont differentiation
Source: PLoS Pathog. 2024 Apr 26;20(4):e1011906. doi: 10.1371/journal.ppat.1011906 (PMC11078545; doi:10.1371/journal.ppat.1011906)

# Supplemental Fig. 1.

—●— Asexual growth

—▲— Gamont maturation/ survival

—◆— Sexual differentiation

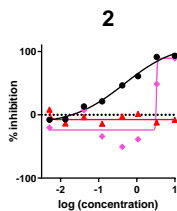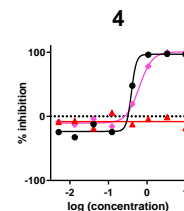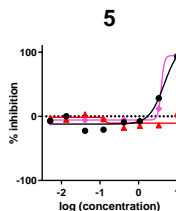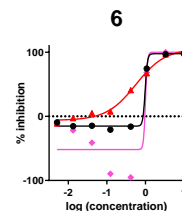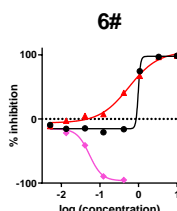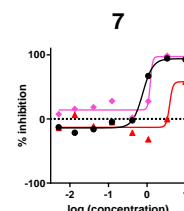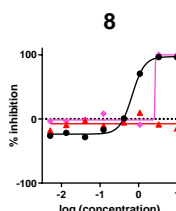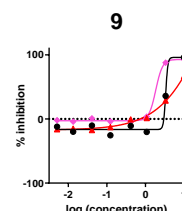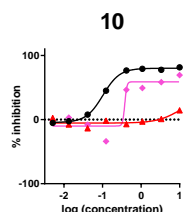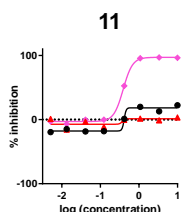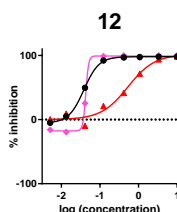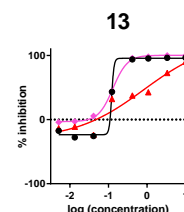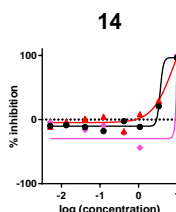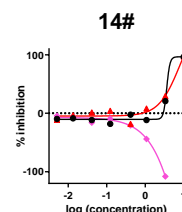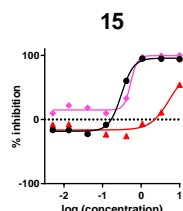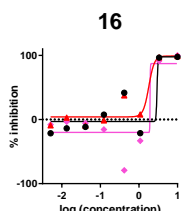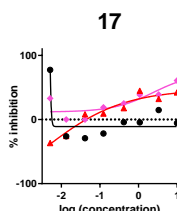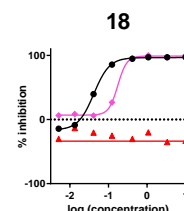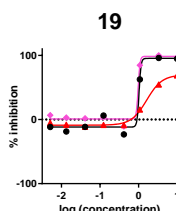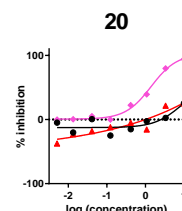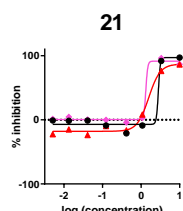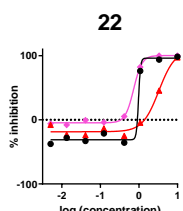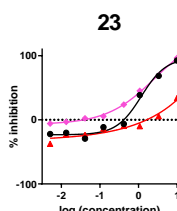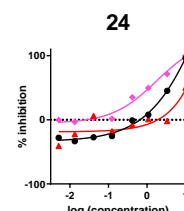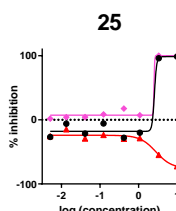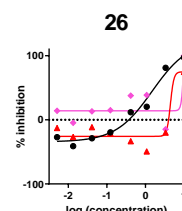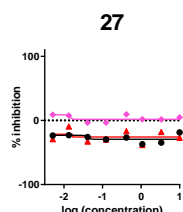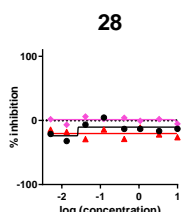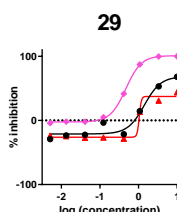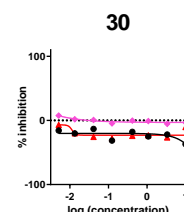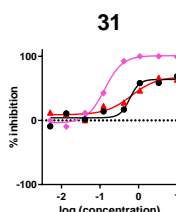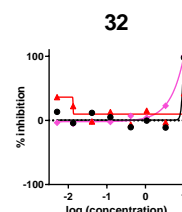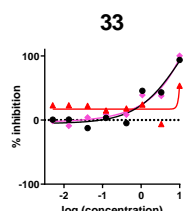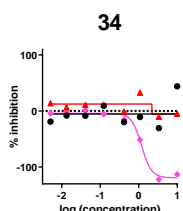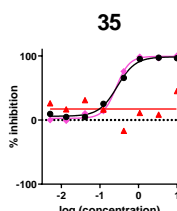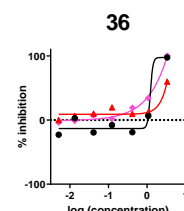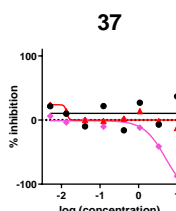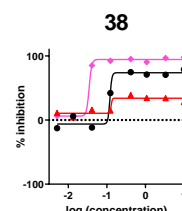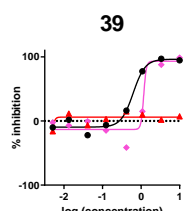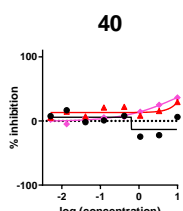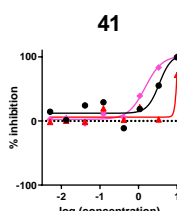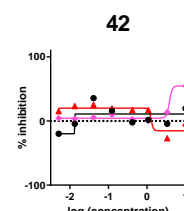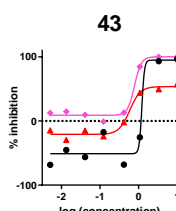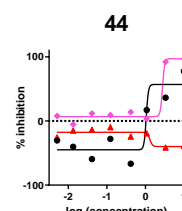

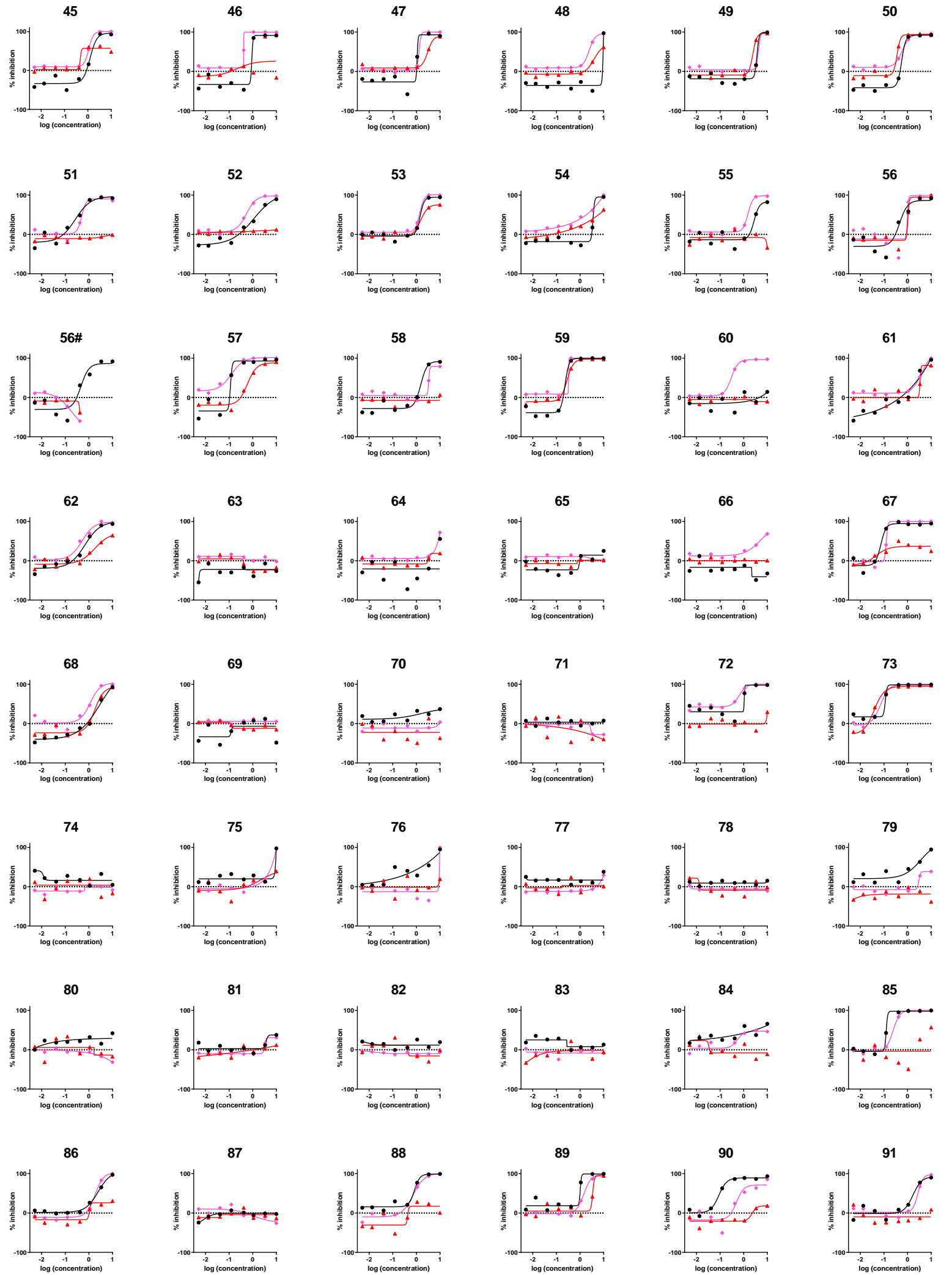

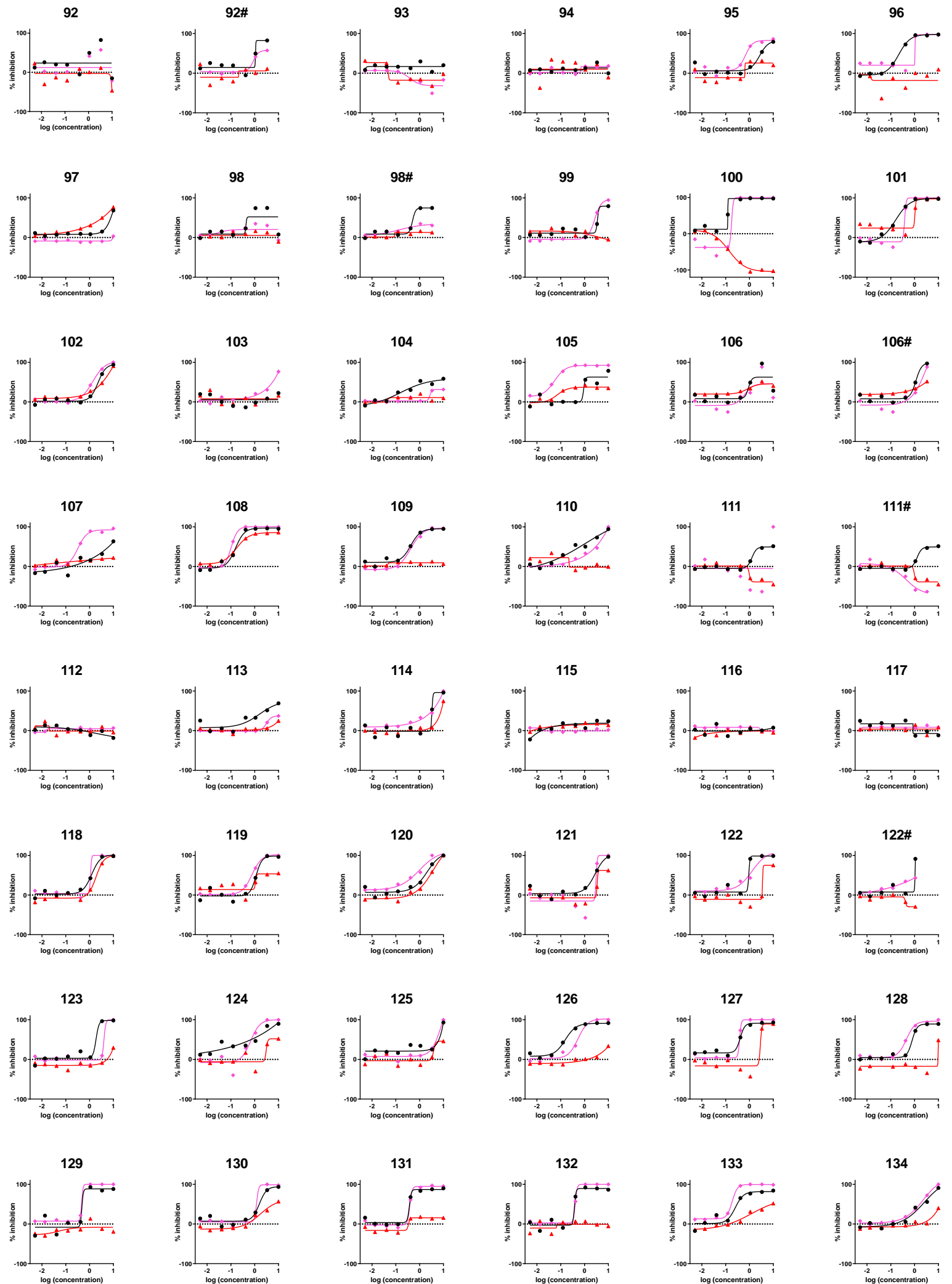

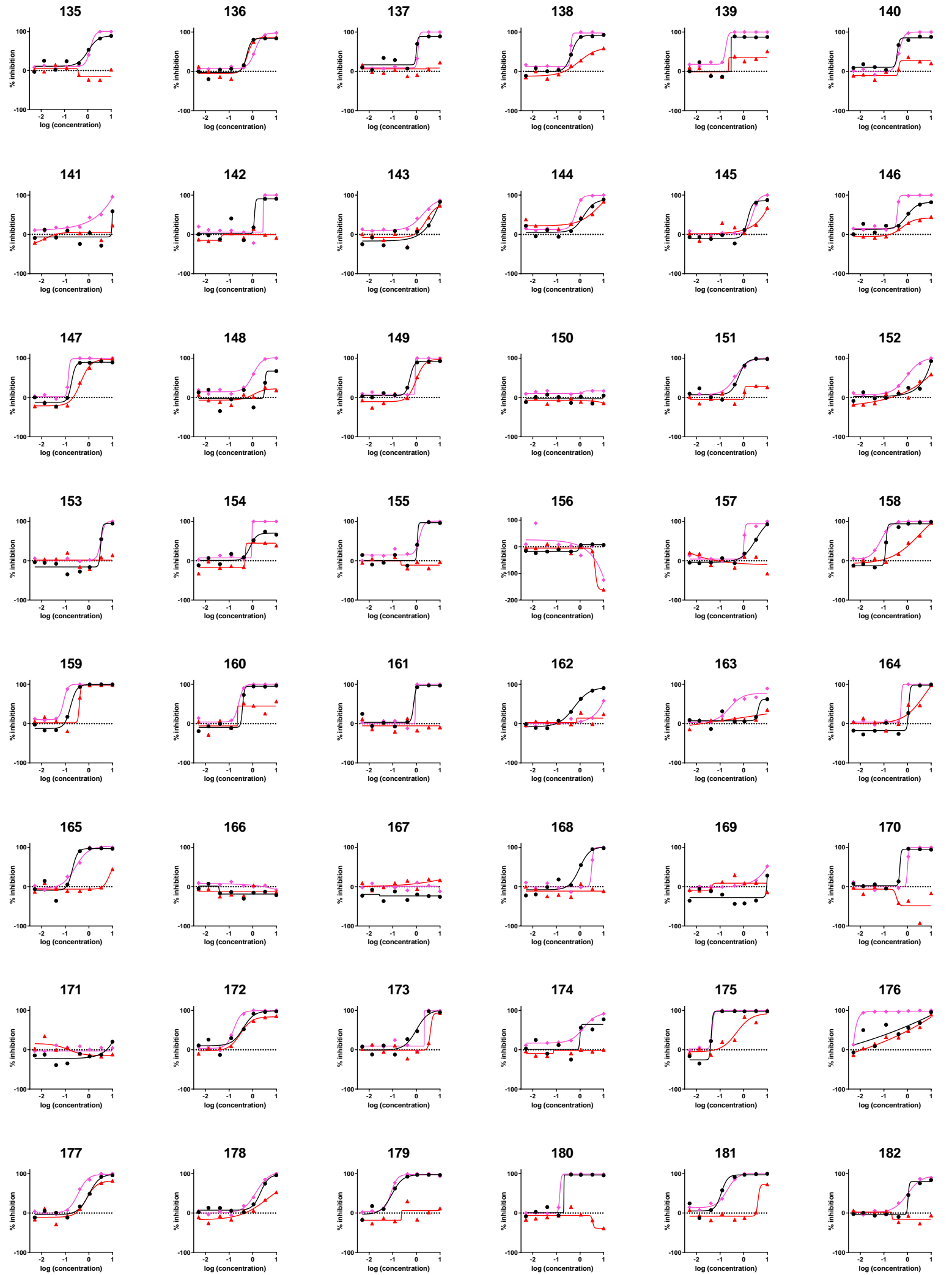

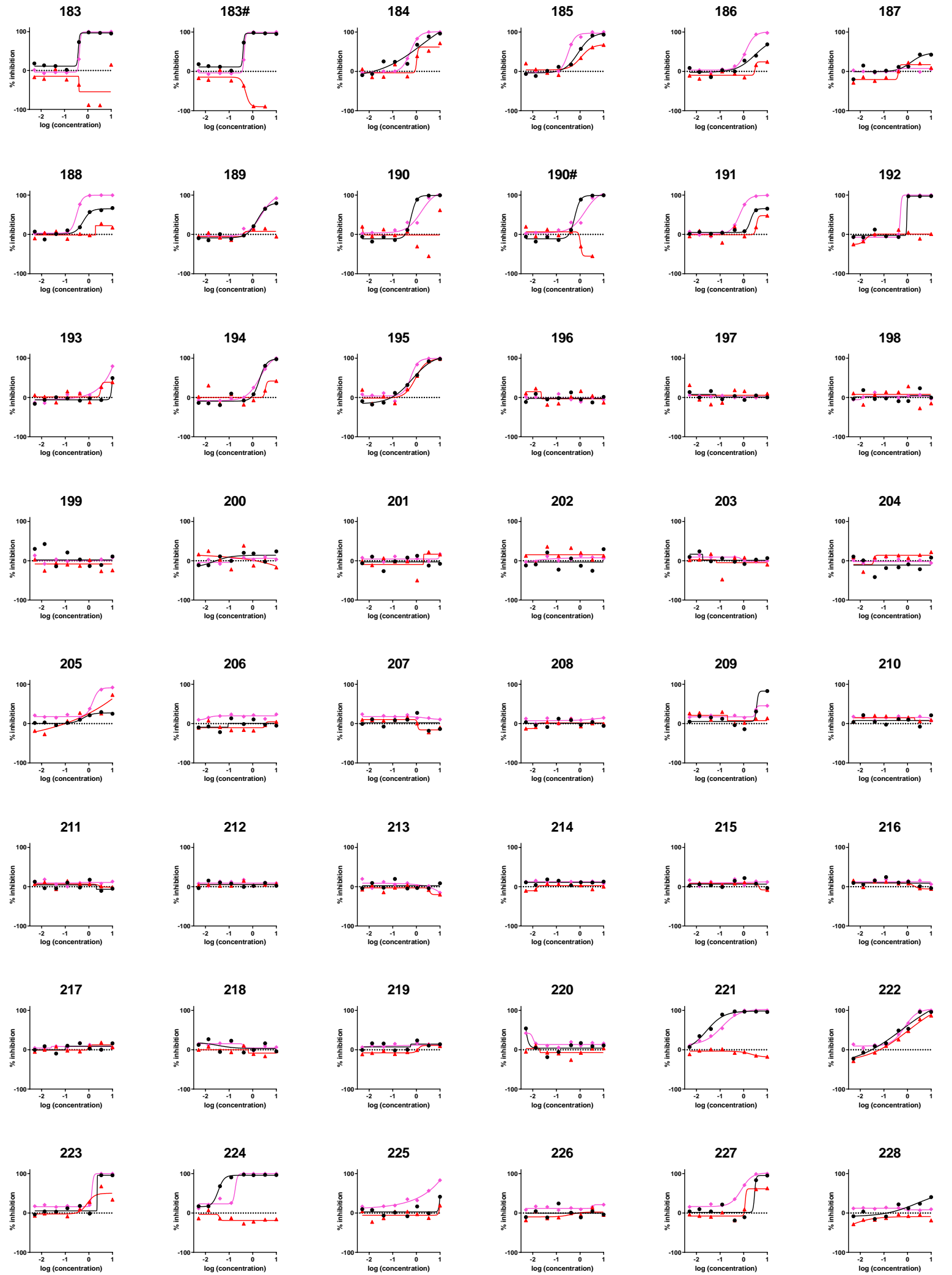

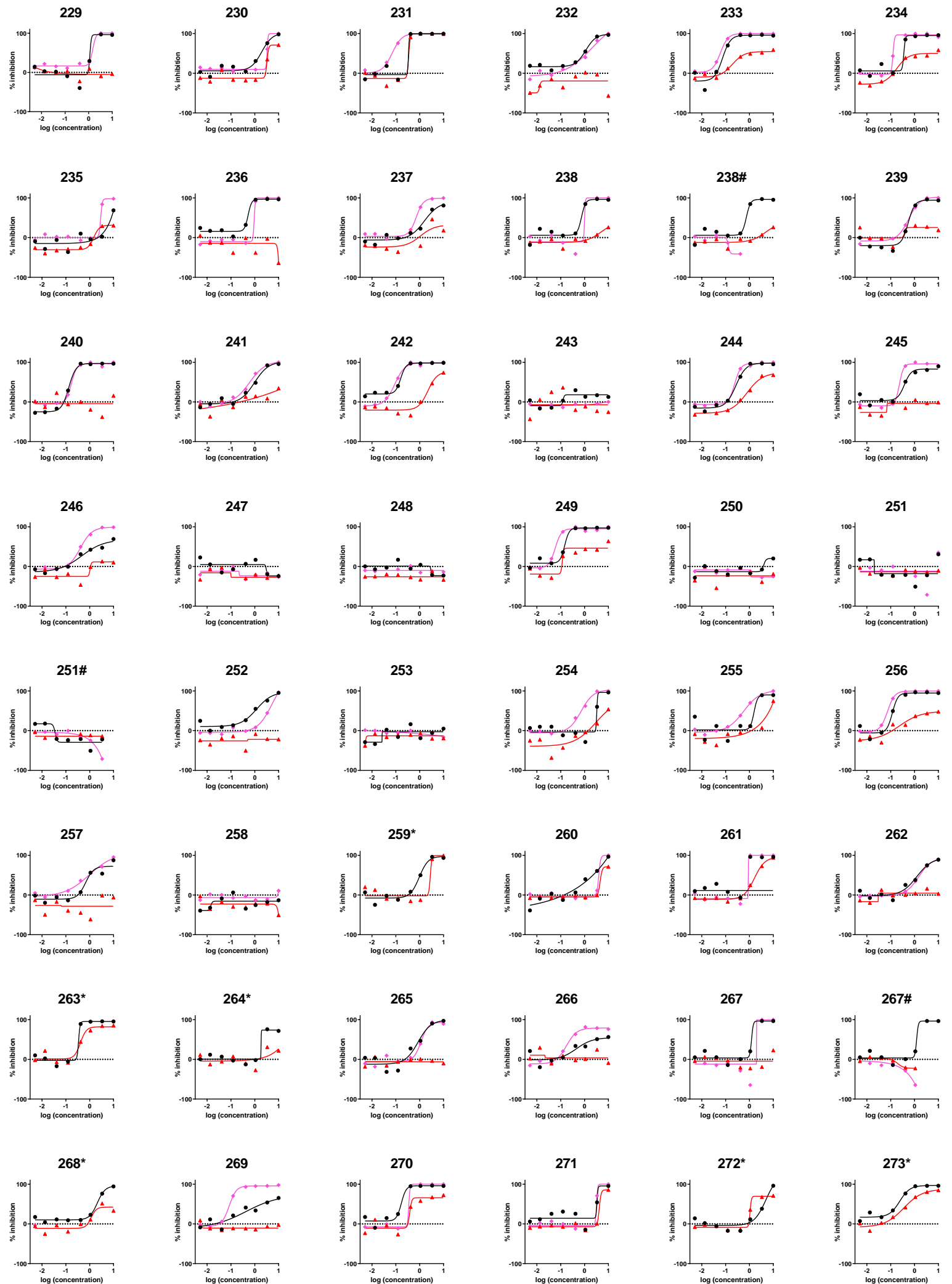

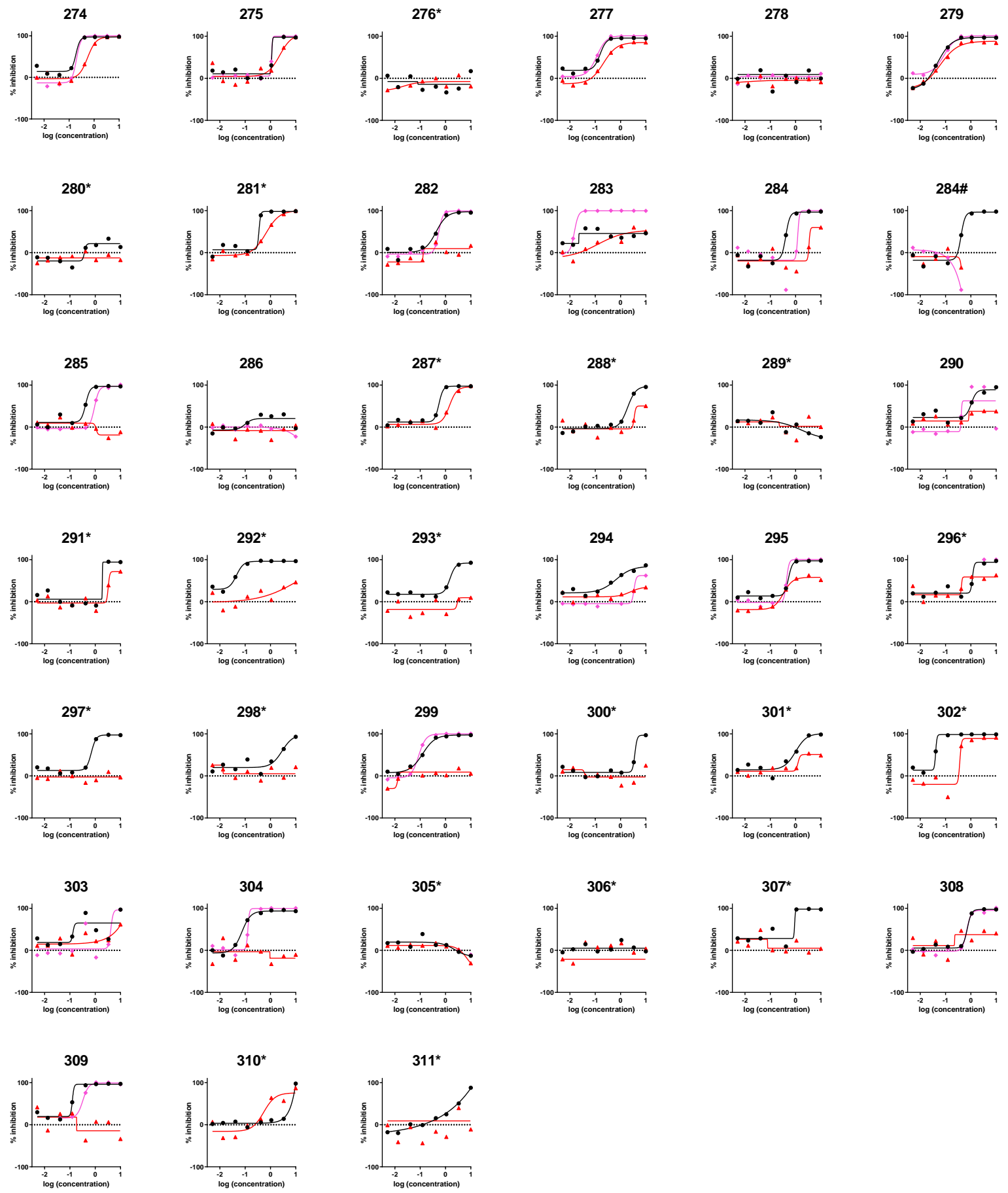

Supplement: S1 Fig — 8-point dose response curves of all the primary screening hits in all 4 readouts. IDs correspond to the compound ids from the first column of S1 Table. Duplicate curves (marked with “#”) are included for compounds that induce sexual differentiation or development in a dose dependent manner at a concentration range that is not highly inhibitory to asexual replication. For the duplicate curves, relevant sexual stage readouts are excluded for high asexual stage inhibitory concentrations. IDs marked with asterisks (*) are compounds without a determined dose response in one of the readouts due to imaging quality error. (PDF) [file ppat.1011906.s007.pdf]

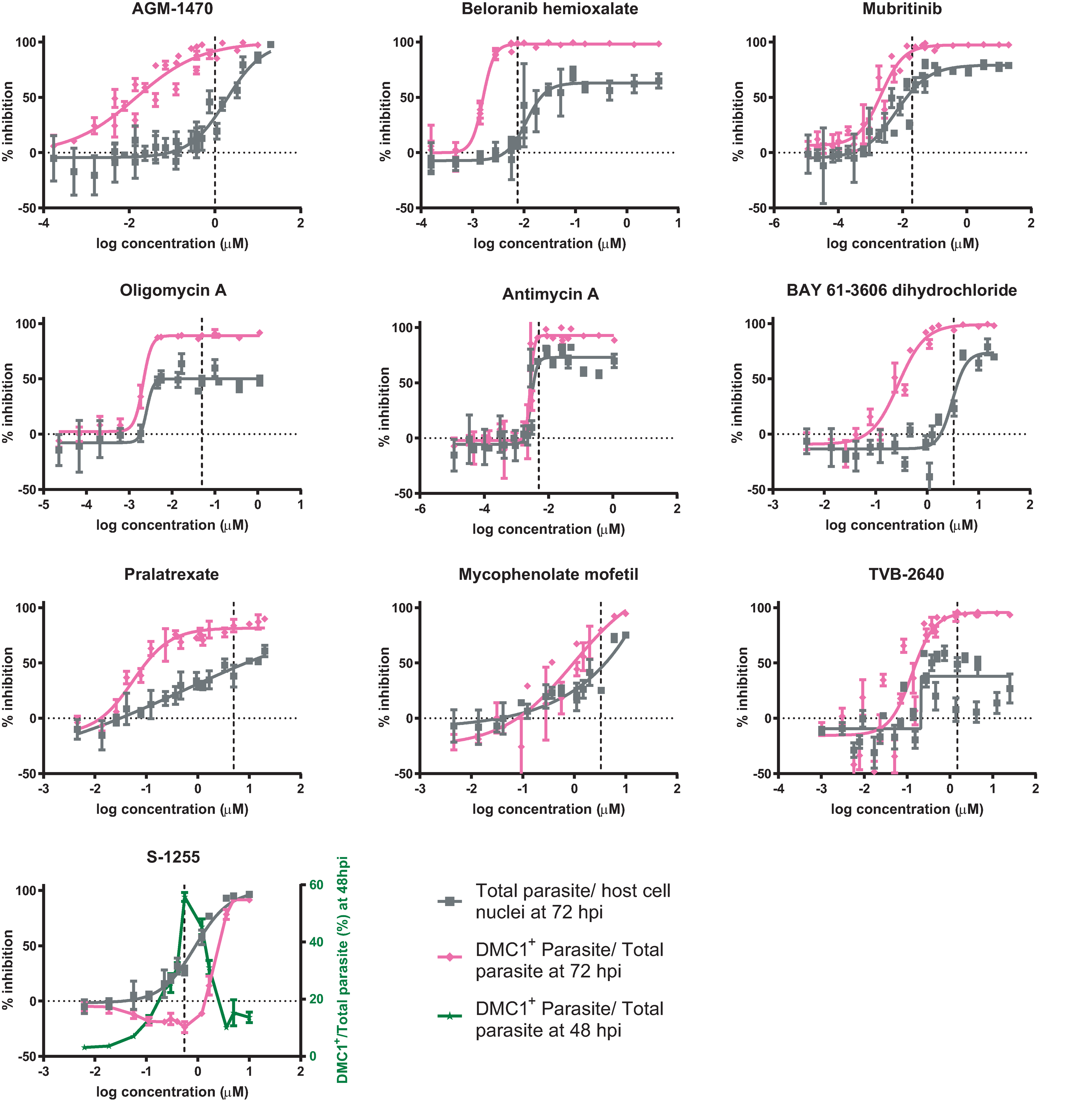

Supplement: S2 Fig — Purchased or resupplied differentiation inhibitors were added at 3 hpi and imaged at 72 hpi (and 48 hpi also for the inducer). Partially overlapping concentration ranges were tested for each of them in 2 to 3 biological replicates. Each point and error bar denotes mean and standard deviation for readings from 4 separate wells for each concentration in each biological replicate. The curves are fitted using the “log(inhibitor) vs. response—Variable slope (four parameters)” model, except for the DMC1 readouts of the macrogamont inducer, where the curve is just a connection between points to highlight the biphasic response. The dotted reference line in the Y axis denotes the concentration selected for RNA-seq experiments. (TIF) [file ppat.1011906.s008.tif]

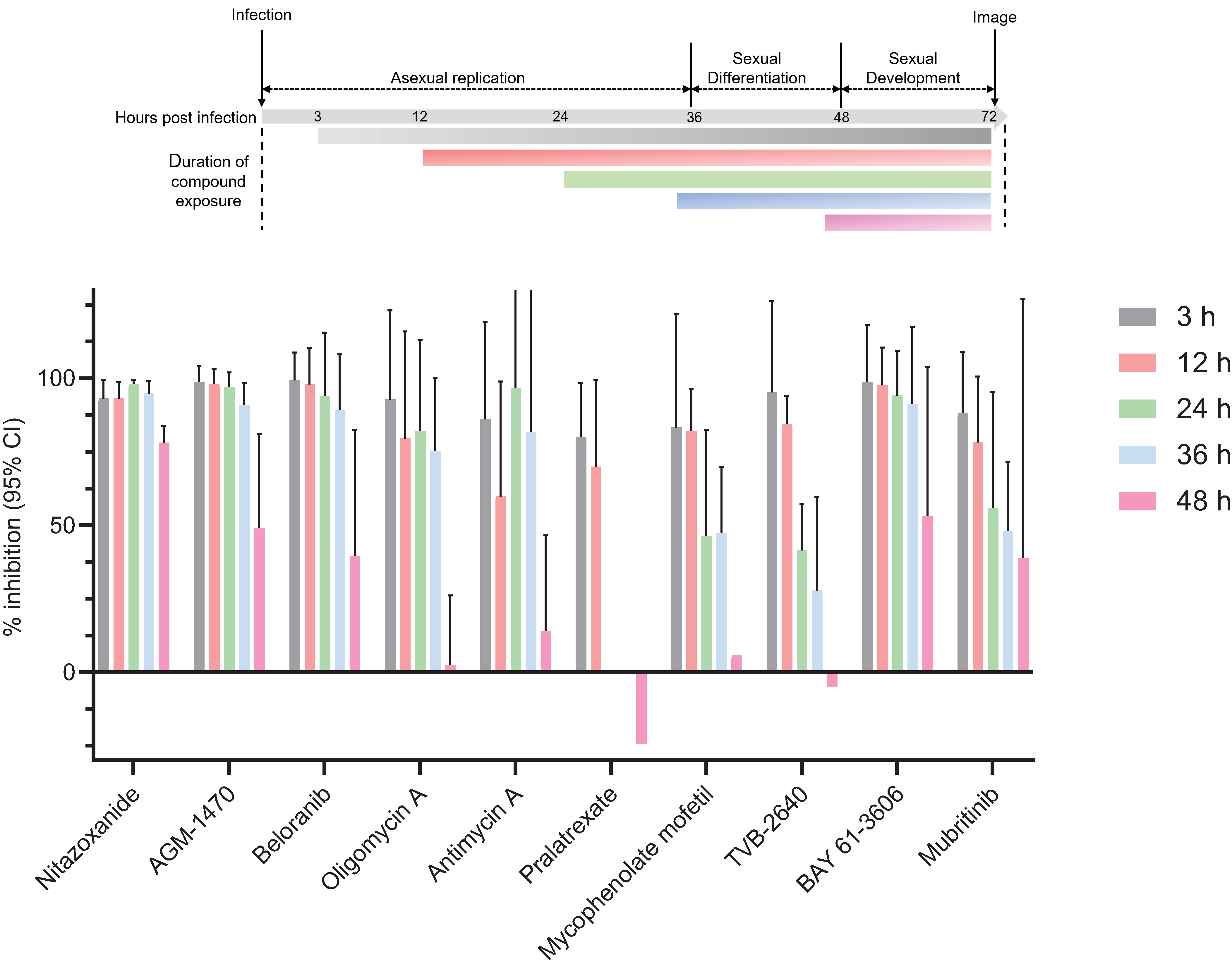

Supplement: S3 Fig — Compounds were added at the indicated time points at the optimal dose to selectively inhibit differentiation, and percent inhibition of macrogamont differentiation (i.e. percent of DMC1+ parasites) was determined at 72 hpi. Data for 2.7 μM nitazoxanide are shown, which was the highest non-toxic concentration tested. (TIF) [file ppat.1011906.s009.tif]

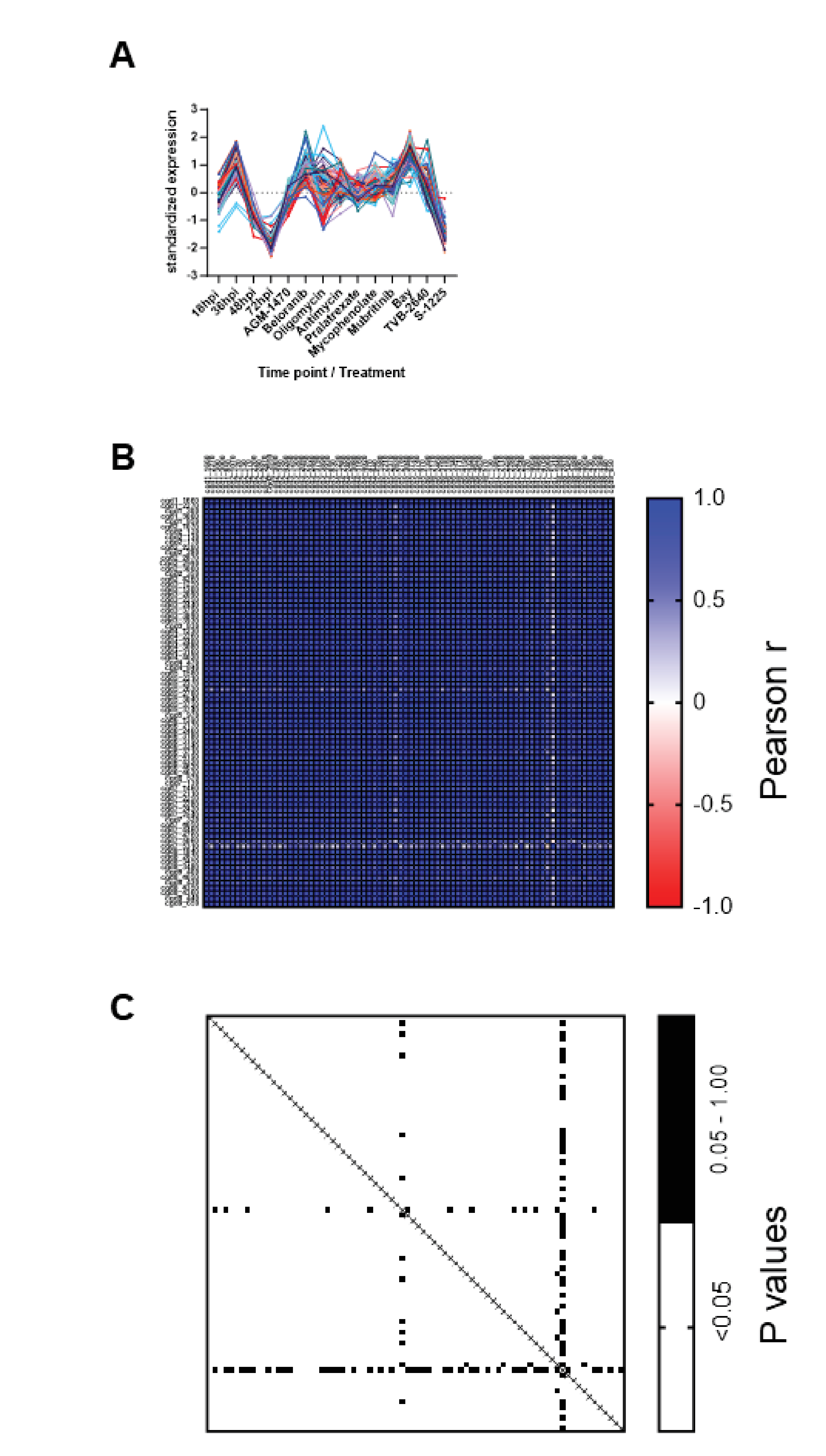

Supplement: S4 Fig — A) Standardized expression of 78 ribosomal protein genes across multiple time points post infection or with compound treatment at 48 hours. Each color represents a gene. Expression levels were normalized by taking the average of expression values for each condition, and then z normalizing it from the expression values across all conditions. B) Corelation matrix of 78 ribosomal protein gene standardized expression level. Note the high degree of correlation among most of the genes. C) Matrix of P values of the corelations shown in panel B. Most of the correlations are statistically significant at α = 0.05. (TIF) [file ppat.1011906.s010.tif]
