# Supplementary material for: Cryptosporidium life cycle small molecule probing implicates translational repression and an Apetala 2 transcription factor in macrogamont differentiation
Source: PLoS Pathog. 2024 Apr 26;20(4):e1011906. doi: 10.1371/journal.ppat.1011906 (PMC11078545; doi:10.1371/journal.ppat.1011906)
Supplement: S1 Table — Number of RNA-seq reads aligned to the human and C. parvum genomes. (DOCX) [file ppat.1011906.s011.docx]

**Supplemental Table 1: Number of aligned sequence reads per sample.**

| Sample | Aligned reads to *C. parvum* genome | Aligned reads to human genome | Percent aligned to *C. parvum* genome | Percent aligned to human genome |
| --- | --- | --- | --- | --- |
| TVB-2640 | 89012 | 21207769 | 0.41796 | 99.58204 |
| TVB-2640 | 398500 | 18929396 | 2.061787 | 97.93821 |
| Beloranib hemioxalate | 91117 | 14583084 | 0.620933 | 99.37907 |
| Beloranib hemioxalate | 552117 | 19454021 | 2.759738 | 97.24026 |
| BAY 61-3606 | 150117 | 19234208 | 0.774425 | 99.22558 |
| BAY 61-3606 | 251870 | 16165615 | 1.534157 | 98.46584 |
| Pralatrexate | 243269 | 15433235 | 1.551806 | 98.44819 |
| Mycophenolate mofetil | 257277 | 23283225 | 1.092912 | 98.90709 |
| Mycophenolate mofetil | 397570 | 16500612 | 2.352738 | 97.64726 |
| Mubritinib | 119264 | 18838548 | 0.629102 | 99.3709 |
| Mubritinib | 128162 | 19117418 | 0.66593 | 99.33407 |
| oligomycin | 81649 | 18853584 | 0.431201 | 99.5688 |
| oligomycin | 132817 | 18338673 | 0.719038 | 99.28096 |
| Antimycin A | 29494 | 14290416 | 0.205965 | 99.79404 |
| Antimycin A | 31028 | 11632588 | 0.266024 | 99.73398 |
| AGM-1470 | 744008 | 23750411 | 3.037459 | 96.96254 |
| AGM-1470 | 393459 | 13474766 | 2.837126 | 97.16287 |
| 48h control 3 | 763848 | 18421278 | 3.981459 | 96.01854 |
| 48h control 2 | 881320 | 17595960 | 4.76975 | 95.23025 |
| 48h control 1 | 341508 | 6751954 | 4.814405 | 95.18559 |
| 18h control 1 | 197351 | 14644283 | 1.329712 | 98.67029 |
| 18h control 2 | 879219 | 16486103 | 5.063073 | 94.93693 |
| 36h control 1 | 678472 | 14406925 | 4.497542 | 95.50246 |
| 36 h control 2 | 1667680 | 15933653 | 9.474737 | 90.52526 |
| 72h control 1 | 219436 | 23041086 | 0.943384 | 99.05662 |
| 72h control 2 | 164540 | 16960315 | 0.960826 | 99.03917 |
| S-1225 | 64414 | 13422511 | 0.477603 | 99.5224 |
| S-1225 | 59269 | 12023862 | 0.49051 | 99.50949 |
