# Supplementary material for: Cryptosporidium life cycle small molecule probing implicates translational repression and an Apetala 2 transcription factor in macrogamont differentiation
Source: PLoS Pathog. 2024 Apr 26;20(4):e1011906. doi: 10.1371/journal.ppat.1011906 (PMC11078545; doi:10.1371/journal.ppat.1011906)
Supplement: Attachment — Submitted filename: response to reviews.docx [file ppat.1011906.s012.docx]

Part I - Summary

Please use this section to discuss strengths/weaknesses of study, novelty/significance, general execution and scholarship.

Reviewer #1: Hasan and colleagues describe the screen of a 12,000-compound library to identify compounds which interfere with C. parvum gamete differentiation in culture. This approach is novel and extends the power of high-throughput screening to the rarely investigated sexual phase of the Cryptosporidium life cycle. Since sexual differentiation is an obligatory phase of the life cycle, Hasan et al. logically argue that inhibitors of gamete differentiation could serve as chemical probes and lead to the discovery of new therapeutic compounds. The manuscript is generally well written and informative.

Reviewer #2: The authors have generated a novel high-throughput small molecule screen that permits them to identify compounds that specifically target the sexual differentiation/gamete formation portion of the Cryptosporidium parvum lifecycle. Cryptosporidium is a significant global human and veterinary diarrheal pathogen that is most prevalent in the very young and immunosuppressed. Currently there is only one approved therapeutic, a drug that targets all life cycle stages, but unfortunately is not approved for use in the most affected populations. The appeal of therapeutic compounds that target this developmental stage is the fact that there is still a bit of controversy as to whether or not Cryptosporidium is obligately sexual in each ~72 hour portion of its life cycle both in vivo and in vitro. Recent work from the Striepen lab revealed a lifecyle for Cryptosporidium that differs from the previously described lifecycle in which only a portion of the parasites enter the sexual cycle. If, as is now appears to be the case, all parasites terminally differentiate into sexual forms in order for the parasite to replicate and complete the lifecycle, a compound that could target this stage could halt infection. Such a compound, as the authors point out, would also be very useful in the laboratory as well and could definitively prove if there is any asexual replication beyond the demonstrated 3 cycles prior to gamete formation. Additionally, as a result of the experiments performed in this work, there is now a list of 65 co-regulated genes that are specifically associated with sexual differentiation, primarily macrogamont formation. Not only are these useful markers, but analyses of their function provided insights into the biology of sexual differentiation in Cryptosporidium and also, possibly gene regulation. This study has a solid experimental design and the experiments are rigorous. I take exception to a few of the interpretations of the results but overall this work represents a significant step forward on several fronts (screens for compounds that affect a different life cycle stage than the one normally targeted in drug screen and the insights gained from the follow-up on the patterns of altered gene RNA expression in parasites affected by particular compounds). The manuscript could be clearer throughout that it is macrogamont differentiation that is being studied and not "sexual differentiation" writ large.

**We thank both of the reviewers for carefully reviewing the manuscript and their overall favorable critiques of our manuscript. Specific issues raised by each reviewer are addressed below. We have addressed all of the critiques as highlighted below in bold purple and believe that the manuscript is much improved as a result.**

________________________________________

Part II – Major Issues: Key Experiments Required for Acceptance

Please use this section to detail the key new experiments or modifications of existing experiments that should be absolutely required to validate study conclusions.

Generally, there should be no more than 3 such required experiments or major modifications for a "Major Revision" recommendation. If more than 3 experiments are necessary to validate the study conclusions, then you are encouraged to recommend "Reject".

Reviewer #1: On line 222 it is stated that the experiment was duplicated, but the Methods section does not describe how duplication was performed. It does make a difference if 2 wells from a same plate were exposed to a compound or the entire experiment was performed with truly independent replicates. The “biological replicate” implies the latter.

**We have performed independent biological replicates. Total RNA samples for each replicate of the same condition reported in the manuscript were collected during separate weeks. Library preparation and sequencing were performed together in batches. We have modified line 222 (now line 245: “independent biological replicates”) and included a sentence clarifying this in lines 569-570 of the methods.**

The statement that the expression of ribosomal proteins is correlated is not apparent from Fig. 4E. A correlation matrix might be a better way to illustrate this point and a statistical test is needed.

**Thank you for the suggestion. We now have added supplemental figure 4 (referred to in line 341) that shows the coregulated expression of ribosomal proteins across treatments and time points, a correlation matrix between their expression, and the P values associated with the correlation coefficients.**

Reviewer #2: I have two concerns:

1) There is the assumption in the manuscript that if the levels of mRNAs for ribosomal proteins decline that this is akin to a state of reduced translation. This may very well be the case, but reduced translation is not explicitly examined or proven. We do not know how long the messages and the proteins they encode are stable. I suggest that the author either prove this point, or, tone down the interpretation to the proven reduced levels of transcription for particular categories of genes. Either way, the finding is significant and as the authors point out, the finding is similar to observations with other apicomplexans. This is surprising given the evolutionary distance between the members of this phylum.

**We apologize for not making this point clearer, but we previously demonstrated overall repression in protein translation using OP-puromycin incorporation, followed by fluorescent labeling with CLICK chemistry and epifluorescence microscopy. Representative images are shown in Figure 4C and quantification of protein translation (OP-puromycin incorporation) vs time is shown in 4D. To clarify, we have now highlighted this by discussing these data under a separate titled subsection (lines 293-320) and modifying the discussion (lines 444-448).**

2) The upstream motif analysis for putative regulatory elements in the coordinately regulated ribosomal protein genes generated a result that is contradictory to a previous result, Oberstaller et al., BMC Genomics 2013 14:516. In the prior analysis, C. parvum ribosomal protein putative promoter regions were found to be enriched for E2F-like and GAGA motifs, contrary to the motifs found in P. falciparum. The major difference between the two analyses is the the definition of upstream region. In the 2013 analysis only upstream regions beginning with the CDS "ATG" that did not encroach into a neighboring gene on either strand were used, and in the analysis reported here, 1 kb of upstream sequence was used. 1 kb almost always overlaps with another gene given the density of this tiny genome. A newly annotated C. parvum genome sequence that includes annotated UTRs was just released by my group and it will permit the best possible analysis since the transcription start site for most transcripts is now known and the UTRs are annotated. See: Genbank nucleotide assembly GCA_035232765.1 and bioRxiv 10.1101/2023.06.13.544219v1.full.pdf for details. It would be ideal to perform this analysis again with the more defined upstream region and also to examine the expression profiles for the several E2F and DP1 transcription factors. The results might change your title.

**Thank you for pointing this out. We have reanalyzed the promoter sequences after trimming off the 5’UTR regions annotated in the latest version of the *C. parvum* IOWA II genome available in the Cryptodb.org server. We already detected before that 1kb sequence around the coding region often encroaches into neighboring genes. We removed such parts from the promoters by removing any coding region of neighboring genes from the 1kb region, as mentioned in lines 368 and 606-608. That is why the promoter sequences are not always 1kb, but lower than that. We have included the UTR trimming procedure in the materials and methods section at line 607-608**

**Importantly, the motif that we reported has a high degree of similarity to the mentioned GAGA motif from Oberstaller et al., BMC Genomics 2013 14:516 (reference 23). The reverse complement of the reported BYGTCTC motif is GAGACRV. The DREME tool searches the motif at both strands of the provided sequences, and the motif that we mentioned was just the reverse complement of the previously reported motif.**

**We now have re-run the same analysis using the UTR trimmed promoter sequences. It found the enrichment of a very similar motif as we reported before. We have modified figure 6 with the new results and also discussed the previous data from Oberstaller et al., BMC Genomics 2013 14:516 in the result subsection at line 348.**

________________________________________

Part III – Minor Issues: Editorial and Data Presentation Modifications

Please use this section for editorial suggestions as well as relatively minor modifications of existing data that would enhance clarity.

Reviewer #1: Given the essential role the immunofluorescent detection of DMC1 plays in the assay, I was wondering about the absence of an uninfected control monolayer in Fig. 1A and 1B. Further regarding Fig. 1A, if the lectin stains any life cycle stage and anti DMC1 antibody reacts with macrogamonts, why do the two signal not overlap in the 72 h merged image? If the original micrographs indicate otherwise, perhaps this should be stated in the text or the legend.

**Specificity of the DMC1 staining is indicated by negative staining at early time points in infected monolayers (see Fig 1A 48 hpi), so no uninfected control monolayers are shown. That said, the screening assay done in 384-well plates includes a background subtraction step in the data handling, with background measured from staining of uninfected wells on every assay plate. For clarity, we have added zoomed-in versions of the 72 hpi images of lectin and DMC1 staining to Fig 1A that show localization of DMC1 to lectin-labeled parasite vacuoles.**

Many blue dots in Fig. 1D are within the 2 SD boundary and yet, according to the legend, were selected for follow-up screens. Why? Also according to the legend, the y axis in 1D is in SD deviation, but the axis label in the figure says % inhibition. Please clarify.

**It is because we used data from multiple readouts to select our hits, but only pan-inhibitors were expected to be a hit in every assay readout. For example, hits classified as differentiation inhibitors by definition had no effect in the assay readout for asexual growth inhibition. Blue dots shown within 2SD for a given assay readout means that the compound was not a hit in that readout but was detected as a hit in at least two other assay readouts. We have further clarified this in lines 175 to 184. The label of the y-axis in figure 1D and meaning of “dot being blue” is further clarified in the figure legend.**

Fig. 3E bar graph, please indicate what constitutes 100%; percentage of what?

**These are the percentages of genes with altered expression profiles from each gene class (unresolved, female-specific, and asexual-specific). We included this graph to highlight** **that higher ratios of the stage-specific genes are dysregulated compared to the unresolved class. The explanation is included in the figure legend.**

Line 528; Please indicate why for the RNA-Seq analysis cell monolayer were infected with different oocyst doses and whether this experimental design introduces an uncontrolled variable, namely oocyst:host cell ratio.

**The reason behind this was to attain an optimized level of infection at the time of sample collection. Insufficient infection would result in fewer parasite gene reads in the RNA-seq whereas too high infection would destroy the host cell monolayer. For samples for later time-points, the initial MOI was kept low because the parasites would get the opportunity to replicate longer and reach an optimum level of infection during sample collection. The explanation is added at lines 565-567. We note that our data demonstrate the expected effect of the compounds despite this variable, as validated using the publicly available RNA-seq data from Tandel, et al.**

Line 310 onward describing the relevance of energy metabolism should probably move to the Discussion. Redundant text in the Discussion, such as lines 388-401 should be removed.

**We moved this section to the discussion. Thanks.**

Line 128, “..but were not visible..” (verb missing)

**Fixed. Thanks.**

Line 136; Do you mean “sexual” differentiation?

**Fixed. Thanks.**

Line 157; “.. that detectably modulated..”

**Fixed. Thanks.**

Lines 285, 309 and Fig. 4E legend; Do you mean “differentially regulated” as opposed to dysregulated? Dysregulation implies an anomaly.

**Fixed. Thanks.**

Reviewer #2: 1. mRNA-seq and RNA-seq both appear throughout the manuscript and this was a bit confusing. I had the sense, perhaps incorrectly that mRNA-seq was being used because a poly-A step is included in the library prep and the focus was on protein encoding genes. However Cryptosporidium has a lot of ncRNAs and most have poly-A tails. If it is the author's intent to make a distinction, please explicitly state how each term will be used.

**We thank the reviewer for this comment and have changed “mRNA-seq” to “RNA-seq” throughout to avoid confusion.**

2. Please state the quantity of reads generated and the percentage of Cryptosporidium transcripts for each library. This can be a supplementary table. The TPM numbers for Cryptosporidium in Supplementary file 2 are decent, but they not high. This is not a surprise. Did you have a cutoff for expression levels to be able to perform the expression analyses? it is not clear from the methods. It appears that NovaSeq was used, is this correct?

**The suggested supplemental Table was added, referred to in line 246. We did not use a cutoff for expression levels for our analysis. The sequencer information is added at line 577.**

3. The methods state that 1 kb upstream was analyzed for motifs but in Supplementary file 6 many shorter promoter lengths are listed. Which sequence was used? 1 kb or the lengths in file 6?

**1kb upstream sequence of the coding region often encroaches into neighboring genes. We removed such parts from the promoters by removing any coding region of neighboring genes from the 1kb region, as mentioned in lines 344 and 368. That is why the promoter sequences are not always 1kb, but lower than that. In this revised version, we have also trimmed the 5’UTR sequence from the promoter, as now mentioned in the material and method section at line 609-610.**

4. The manuscript does not have a data accessibility section. Have the RNA-seq reads that were generated been deposited in the SRA? Please provide the bioproject and accession numbers. I realize the data for other species come from VEuPathDB.org, but they are not an archive for new data5. A potential item to add to the discussion is the observation in Figure 3C that the number of upregulated genes is surprisingly uniform but the number of down-regulated genes is highly variable. Do the authors have any thoughts on this?

**Bioproject and accession provided at line 616. We do not find the number of upregulated genes to be uniform.**

5. Figure 4A. Please clarify if these data are only for C. parvum

**Mentioned in the figure legend (line 677).**

6. Figure 7 legend, last line, "transcript factors that bind a" without the s on binds

**Thanks. Fixed.**

7. Lines 44-45 translational repression is not experimentally validated

**Translational repression was demonstrated experimentally using OP-puromycin staining as detailed above under major concerns. Data are shown in figure 4C and 4D.**

8. Line 81 " Cryptosporidium sexual differentiation"

**Thanks.**

9. Line 116 mechanism? perhaps pathway or regulation?

**Changed.**

10. Table 1 - Just curious. Given the recent results from the IMPDH knockout in Cryptosporidium why do you think mycophenolate still came through the screen as strongly as it did? perhaps an off target host effect?

**We think it is likely an off-target host effect, as are the effects of other compounds except the MetAP2 inhibitors. Our rationale for this is the numbers of host genes vs. parasite genes with altered expression following compound treatment. Lines 412 to 442 of the discussion describe this (formerly in the results section) with mycophenolate specifically discussed in lines 438-441.**

11. Line 268 all sexually differentiated, or just macrogamonts? I thought the screen could only detect the female gamete.

**This is an excellent point. We agree that our screen can only detect macrogamont differentiation. This line was changed, as was the title. Additional modifications were made throughout the text to draw this distinction.**

12. Line 287 VEuPathDB.org

**Thanks.**

13. Line 327 perhaps move this to the discussion?

**Thank you. We moved this section to the discussion (now lines 412-442) and renumbered the figures appropriately.**

14. Lines 353-354, not necessary. Gene regulation is often regulated combinatorially either by differing numbers of the same TF site or combinations of TF sites or both. please consider rephrasing.

**We rephrased the line with “We hypothesized that this is a transcription regulatory motif and if that is true, majority of the other genes having this motif in their promoter region might have similar expression profiles to RP genes” at line 348-350.**

15. Lines 416-417 and 425, this is not proven, please revise.

**Please see figure 4. Discussed in the response to the first major concern of reviewer 2.**

16. Lines 455-457 I think a reference is missing, these results are not from this study.

**There are no published results looking at in vivo efficacy of *Cryptosporidium* differentiation inhibitors. We were actually referring to mouse experiments that we did with two compounds for which the results were negative and thus unlikely to ever be published. We note that these negative results are uninterpretable, since most compounds are not retained in the small intestine long enough to treat *Cryptosporidium* and specialized PK studies would be needed to determine intestinal compound concentrations. Since most ReFRAME library compounds were developed with a goal of rapid absorption from the GI tract after oral administration, the better long-term approach to testing this is beyond the scope of this study but would be identification and/or synthesis of chemical analogs with the right pharmacokinetic/dynamic characteristics to achieve in vivo efficacy. Since report of “data not shown” is not permitted, we modified this to simply state that “Further in vivo work would help to understand if *Cryptosporidium* sexual differentiation is indeed obligatory….” and then note the caveats associated with negative in vivo studies to clarify that it’s not as simple as just testing some of the identified inhibitors in vivo (lines 485-490).**

17. Line 546 CryptoDB.org

**Thanks.**

18. Line 547 add italics

**Thanks.**

19. Lines 566-574, please define how UTR and promoter regions were determined/are defined

**They are now defined, and reanalysis with the modified sequences are reported.**

20. Should the title of the manuscript make it clearer that the topic is macrogamont differentiation?

**We agree and have changed the title. We have also added clarification throughout the manuscript. There is ample reason to believe that a screen done with a microgamont marker would likely yield different results, which is discussed in lines 499-503.**
